# Using Lung Point-of-care Ultrasound in Suspected COVID-19: Case Series and Proposed Triage Algorithm

**DOI:** 10.5811/cpcem.2020.7.47912

**Published:** 2020-07-16

**Authors:** Nicole M. Duggan, Andrew S. Liteplo, Hamid Shokoohi, Andrew J. Goldsmith

**Affiliations:** * Massachusetts General Hospital, Division of Ultrasound in Emergency Medicine, Department of Emergency Medicine, Boston, Massachusetts; † Brigham and Women’s Hospital, Division of Emergency Ultrasound, Department of Emergency Medicine, Boston, Massachusetts

**Keywords:** lung, ultrasound, POCUS, COVID-19, SARS-CoV-2, coronavirus

## Abstract

**Introduction:**

First detected in December 2019, the severe acute respiratory syndrome coronavirus 2 pandemic upended the global community in a few short months. Diagnostic testing is currently limited in availability, accuracy, and efficiency. Imaging modalities such as chest radiograph (CXR), computed tomography, and lung ultrasound each demonstrate characteristic findings of coronavirus disease 2019 (COVID-19). Lung ultrasound offers benefits over other imaging modalities including portability, cost, reduced exposure of healthcare workers as well as decreased contamination of equipment such as computed tomography scanners.

**Case Series:**

Here we present a case series describing consistent lung ultrasound findings in patients with confirmed COVID-19 despite variable clinical presentations and CXR findings. We discuss a triage algorithm for clinical applicability and utility of lung point-of-care ultrasound in the setting of COVID-19 and advocate for judicious and targeted use of this tool.

**Conclusion:**

Lung point-of-care ultrasound can provide valuable data supporting diagnostic and triage decisions surrounding suspected cases of COVID-19. Prospective studies validating our proposed triage algorithm are warranted.

## INTRODUCTION

Coronavirus disease 2019 (COVID-19), the illness caused by severe acute respiratory syndrome coronavirus 2 (SARS-CoV-2) infection, ranges in presentation from mild cold-like symptoms to hypoxemic respiratory failure.^1^,^2^ As of late-June 2020, there are nearly nine million confirmed cases, and more than 400,000 deaths worldwide attributable to COVID-19.^3^ SARS-CoV-2 infection is confirmed by reverse-transcriptase polymerase chain reaction (RT-PCR). This presents challenges for physicians as testing availability is often limited and results delayed. RT-PCR demonstrates imperfect sensitivity, often requiring multiple tests to confirm a patient’s status.^4^ Additionally RT-PCR cannot predict clinical course or outcomes. Emergency department (ED) physicians caring for patients with suspected COVID-19 often must make quick and consequential clinical decisions. Data that can be rapidly gathered in real-time to support or refute this diagnosis is invaluable.

Characteristic chest computed tomography (CT) and radiograph (CXR) findings are described in COVID-19 particularly in peripheral and posterior lung distributions.^5^–^7^ While the sensitivity of CT for COVID-19 ranges between 86–97%, CXR sensitivity is as low as 59%.^5^,^8^ As a result, CT is proposed as a screening tool for COVID-19 when confirmatory tests are lacking.^4^ CT scans have high accuracy in detecting the presence and severity of lung involvement but logistical challenges such as exposing additional healthcare workers, patients, and the CT scanner itself to the virus limit its utility.

Lung point-of-care ultrasound (POCUS) is crucial for assessing patients with dyspnea in the ED.^9^–^12^ Lung POCUS has higher sensitivity than CXR for detecting viral and bacterial pneumonia.^10^–^13^ In limited reports on the use of lung POCUS in COVID-19, findings appear similar to features typically seen in viral and bacterial pneumonia or interstitial syndrome.^14^–^16^ Here we present a series of cases of lung POCUS findings in patients with confirmed COVID-19. Given our experience, we propose a five-tier model for responsible and clinically applicable use of lung POCUS in patients with suspected COVID-19.

## CASE SERIES

### Case 1

**Image 1:**
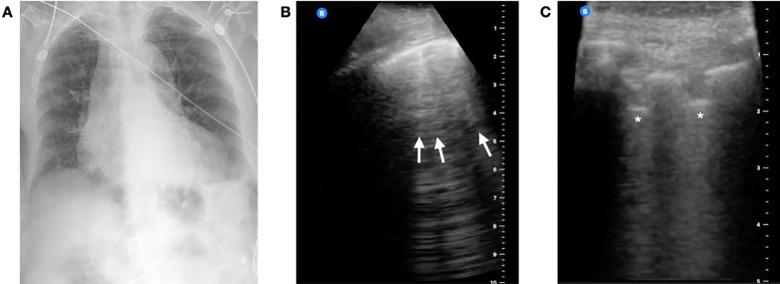
Case 1 imaging findings: (A) Anterior-posterior chest radiograph was read as negative, (B) Lung point-of-care ultrasound revealed B-lines anteriorly (arrows), (C) and an irregular pleural line with subpleural consolidations posteriorly (asterisks).

A 93-year-old female with a history of atrial fibrillation and congestive heart failure presented from a nursing facility with three days of cough and fevers in acute respiratory distress. On arrival to the ED, her oxygen saturation was 93% on a non-rebreather mask at 15 liters per minute. She was tachypneic with respiratory rates in the mid-30s, tachycardic to 130 beats per minute, and febrile to 102° Fahrenheit. A CXR was performed that was read as negative (Image 1A). A lung POCUS was performed and showed posterior subpleural consolidations (Image 1B) and diffuse B-lines bilaterally (Image 1C). After a conversation with the family about a presumed diagnosis of COVID-19, the patient was confirmed to be do not resuscitate/do not intubate and admitted to the medicine floor on supplemental oxygen. An RT-PCR for SARS-CoV-2 resulted positive the next day.

**CPC-EM Capsule**

What do we already know about this clinical entity?

Coronavirus disease 2019 (COVID-19) has variable presentation and progression. Appropriate triage of these patients is key to minimizing morbidity and mortality.

What makes this presentation of disease reportable?

In each presented case, data provided by point-of-care ultrasound (POCUS) was used to guide clinical care and triage decisions in patients with suspected COVID-19.

What is the major learning point?

COVID-19 demonstrates characteristic lung POCUS findings including an irregular pleural line, B-lines, and subpleural consolidations.

How might this improve emergency medicine practice?

Lung POCUS may be used as an inexpensive and accessible tool for diagnosis and triage in patients with suspected COVID-19 in various settings.

### Case 2

**Image 2:**
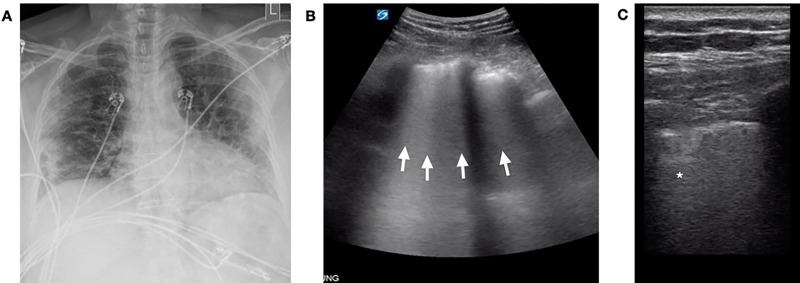
Case 2 imaging findings: (A) Anterior-posterior chest radiograph notable for peripherally distributed bilateral patchy opacities. Lung point-of-care ultrasound was notable for diffuse B-lines coalescing to involve the entire rib space (arrows) laterally (B), while posterior views revealed irregular pleural lines and subpleural consolidations (asterisk, C).

A 66-year-old female with a history of hypertension presented with several weeks of fatigue, fevers, and shortness of breath. On arrival to the ED, she was tachypneic with a respiratory rate in the mid-30s, and an oxygen saturation of 90% on 2 liters of oxygen by nasal cannula. Her vital signs and physical exam were otherwise unremarkable. CXR revealed bilateral diffuse patchy opacities distributed peripherally (Image 2A). A lung POCUS was notable for diffuse bilateral confluent B-lines anteriorly (Image 2B) and an irregular pleural line posteriorly with multiple consolidations (Image 2C). Despite maintaining oxygen saturations between 90–95% on supplemental oxygen, after discussion with the patient and family, the decision was made to pursue early intubation and admission to the intensive care unit (ICU) given her remarkable lung POCUS and expected clinical course. Definitive testing for SARS-CoV-2 in the form of RT-PCR resulted in positive days later. She was successfully extubated on hospital day ten and later transferred to the medical floor in stable condition.

### Case 3

**Image 3:**
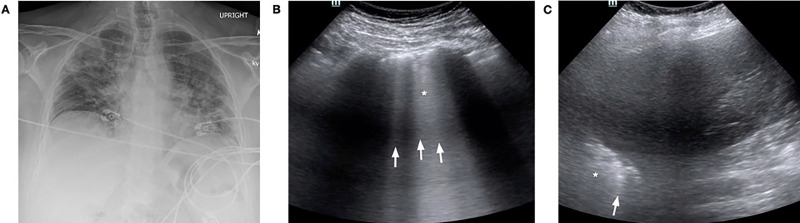
Case 3 imaging findings: (A) Anterior-posterior chest radiograph at the time of presentation to the emergency department demonstrating diffuse peripherally-based bilateral patchy opacities. Lung point-of-care ultrasound in the emergency department was remarkable for confluent B-lines posterior (B, arrows), as well as an irregular pleural line with subpleural consolidations (asterisk). A lateral view (C) in the mid-anterior axillary line showed similar B-lines (arrow) and an irregular pleural line (asterisk).

A 56-year-old female with a history of fibromyalgia, hyperlipidemia, depression, and travel to New York City ten days earlier presented with respiratory distress after one week of progressive fevers, chills, and a dry cough. She presented to urgent care five days prior where vital signs and a CXR at that time were normal. She was sent home with an albuterol inhaler, steroid taper, and instructions to socially isolate. In the ED, she was hypoxic to 70% on room air, which improved to 90–94% on a non-rebreather mask at 15 liters per minute. A CXR showed bilateral diffuse interstitial opacities (Image 3A). A lung POCUS revealed bilateral confluent B-lines, an irregular pleural line, and consolidations in the posterior and lateral fields (Image 3B and 3C). She was emergently intubated for hypoxemic respiratory failure and admitted to the ICU. Definitive testing for SARS-CoV-2 resulted positive the following day. Despite refractory hypoxemia, she was successfully extubated on hospital day sixteeen, and was eventually transferred to a rehabilitation facility.

## DISCUSSION

Here, we describe a case series of SARS-CoV-2-positive patients with variable clinical presentations. We present lung POCUS findings seen in COVID-19, notably B-lines, an irregular pleural line, and subpleural consolidations (Table). Representative clips of these findings are seen in Videos 1 and 2. Given our experience, we propose a five-tier model to guide decision-making for integrating lung POCUS in assessing patients with suspected COVID-19 (Figure 4).

In case one, lung ultrasound aided in the diagnosis of COVID-19. While COVID-19 was suspected from clinical history, CXR was not consistent with clinical findings, and confirmatory RT-PCR result was not immediately available. As is common in these cases, further studies were needed to support the diagnosis and direct clinical care. Lung POCUS confirmed that a bacterial and/or viral pneumonia was most likely and provided physicians with actionable information in a rapid and non-invasive manner.

In areas with high disease prevalence, lung POCUS consistent with bilateral viral or bacterial pneumonia is highly suggestive of COVID-19. In these cases, lung POCUS can serve as a screening tool for suspected viral infection and may obviate the need for CT. This is particularly important in resource-limited settings where CXR, CT, or RT-PCR may not be readily available. CT is more sensitive than CXR for findings of COVID-19, thus it too is suggested as a screening tool. Routine use of CT scan presents challenges beyond limited availability, however. Safely moving a patient with cardiopulmonary instability to the CT scanner is not always feasible and often requires multiple healthcare workers. From an infection control standpoint, exposing additional healthcare workers and the CT scanner to coronavirus increases the risk of disease spread. In our cases, physicians were able to forgo CT scans, thus avoiding unnecessary viral exposure to additional staff, patients, and equipment.

Screening via lung POCUS could occur in many settings, including triage tents, EDs, and under-resourced environments with limited access to other diagnostic studies. It is likely that in many areas, globally, nasal swabs or serologic testing are not available, whereas ultrasounds may be. Given that only a power source is needed to operate, ultrasound machines could have a prominent role in screening for COVID-19 in these settings.

In case two, lung POCUS helped guide decision-making surrounding early intubation and ICU admission (i.e., tier four in Figure). Often the disposition of ED patients can be made on clinical grounds alone. At times, however, imaging can help guide these decisions. When POCUS findings are more prominent than CXR findings, a worse clinical disease could be suspected and upgrades in the level of care initiated. In this case, the patient clearly required admission, but the extent of disease and need for interventions such as intubation were uncertain from exam and CXR alone. Our POCUS and the patient’s borderline respiratory status triggered a meaningful discussion with the patient regarding her expected clinical course. She confirmed her preference for early intubation, and the procedure was performed in a controlled setting. Here, lung POCUS dictated our decision to upgrade the patient’s clinical care.

Similarly, patients being considered for admission could also benefit from lung POCUS (i.e., tier two in Figure). As in case one, since ultrasound is more sensitive than CXR for early pulmonary disease, lung POCUS may reveal findings consistent with COVID-19 when CXR remains negative. For patients who have borderline dispositions, lung POCUS can help lower the threshold for admission. Conversely, if both CXR and ultrasound are negative, providers may feel more comfortable discharging patients home with follow up. Lung POCUS has the potential for high clinical utility in these cases, and the benefits of use likely outweigh risks.

In case three, though ultrasound findings were prominent, they did not affect the patient’s clinical course. In cases where disposition and management are clear, ultrasound may not be necessary (i.e., tier five in Figure), and its use should be carefully considered. As lung POCUS did not change our management, it was likely not worth the additional exposure risk to physicians. Similarly, on the opposite end of the spectrum, lung POCUS may also not be indicated in patients with suspected COVID-19 who are well-appearing, have adequate oxygen saturations, and are otherwise well enough for discharge (i.e., tier one in Figure). Lung POCUS may not contribute to the medical decision-making in these cases, and thus risks of increased viral exposure from performing POCUS likely outweigh the benefits of performing ultrasonography.

In patients who require admission but are stable for the medical floor, ultrasound may also not be indicated (i.e., tier three in Figure). In these patients CXR is likely positive, and clinical symptoms such as dyspnea, tachypnea, or hypoxia support admission. While lung POCUS can always be used to assess for alternative causes of dyspnea, in patients with a clinical history and workup consistent with COVID-19, lung ultrasound likely has limited clinical benefit. Judicious use of lung POCUS is advised in these patients, given the likely limited clinical utility compared to the risks of increased exposure.

## LIMITATIONS

While lung POCUS may provide rapid and actionable clinical data for patients with suspected COVID-19, this imaging modality also has limitations. Though often more sensitive than CXR, lung POCUS findings described here are not specific to COVID-19. These findings are seen in a range of alveolar-interstitial syndromes, thus are not definitively diagnostic of SARS-CoV-2 infection. For cases of suspected COVID-19, our experience and the experience of others suggest lung POCUS may have higher utility than CXR for detecting early disease, though little is known regarding POCUS prognostic capabilities.^14^,^15^ As suggested by others, combining lung POCUS with additional clinical data such as vital signs and serum laboratory results may likely provide the highest clinical utility.^17^–^19^ Further studies focused on diagnostic and prognostic capabilities of lung POCUS in COVID-19 are needed.

An exception to consider in our model is that for any patient, POCUS can be used to identify alternative causes of respiratory distress.^20^ In areas of high disease prevalence for COVID-19, our model can be used to dictate the safe and effective use of lung POCUS in patients under investigation of SARS-CoV-2 infection. For patients with comorbidities or clinical pictures inconsistent with COVID-19; however, cardiac and pulmonary POCUS should be considered to assess for alternative diagnoses. In the era of COVID-19, the risk/benefit ratio of performing POCUS must be carefully considered for each case. Given these limitations, prospective studies assessing our proposed triage algorithm are needed to further assess its utility in clinically undifferentiated patients in a variety of healthcare settings.

Currently, supportive care is the mainstay treatment for COVID-19. As further research identifies targeted treatment algorithms, earlier and more rapid diagnosis may have management implications. In the future, lung POCUS screening may play a role in cases requiring earlier diagnoses, and its utility in assessing patients with suspected COVID-19 should be continuously re-evaluated as this pandemic evolves.

## CONCLUSION

The now-ubiquitous nature of COVID-19 demands a more rapid, safe, and accurate clinical evaluation than CXR, CT, or RT-PCR can currently provide. Lung POCUS offers valuable clinical data to first-line responders when assessing patients with suspected COVID-19. Given risks of exposure to providers and possible device contamination, POCUS is no longer considered risk-free. Though valuable, this tool should be used judiciously and reserved for cases in which it may alter patients’ clinical course. Providers must be thoughtful about the cases in which we pursue POCUS and ensure our efforts will confer the highest clinical benefit while minimizing risk overall.

## Supplementary Information

Video 1 Ultrasound clip demonstrating coalescent and individual B-lines in a patient with coronavirus disease 2019. This image was obtained using a curvilinear transducer positioned at the anterior chest wall.

Video 2 Ultrasound clip demonstrating an irregular pleural line and subpleural consolidations in a patient with coronavirus disease 2019. This image was obtained using a linear transducer positioned at the posterior thoracic wall.

## Figures and Tables

**Figure:**
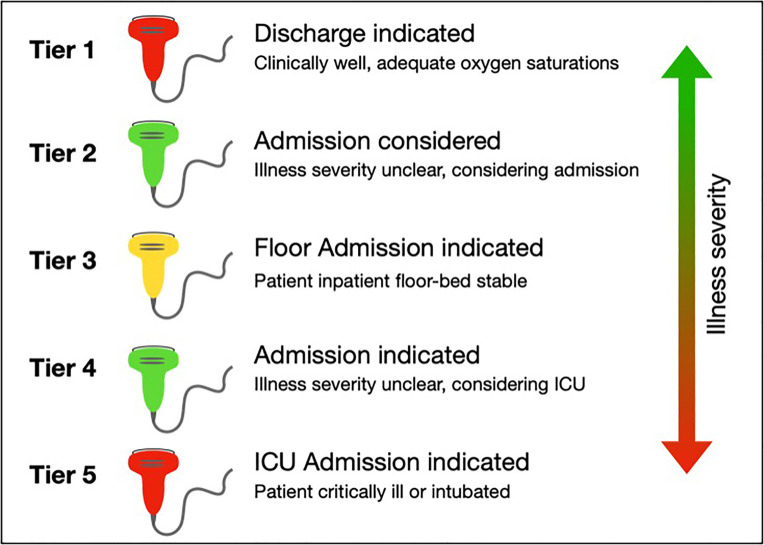
Proposed triage model of lung point-of-care ultrasound (POCUS) indications when evaluating patients with suspected coronavirus disease 2019. In tier one, patients for whom discharge is indicated, lung POCUS likely does not contribute to clinical decision making thus has limited utility (indicated by the red probe). In tier two, for patients who do not clearly meet admission criteria, lung POCUS may reveal increased severity of disease and indicate the need for admission. Thus, has high utility potential (indicated by the green probe). For patients who meet admission criteria but are stable for the medical floor, lung POCUS may contribute to clinical decision making and should be used at the discretion of the emergency department provider (i.e., tier three, indeterminate clinical utility indicated by the yellow probe). For patients who should be admitted but may require advanced interventions such as intubation or intensive care unit (ICU) admission, lung POCUS likely could help guide clinical decision-making (i.e., tier four). In patients who are critically ill and immediately warrant ICU admission, lung POCUS will rarely change the clinical course and is often not indicated (i.e., tier five).

**Table:** Summary comparison of findings in chest radiograph and lung point-of-care ultrasound (POCUS) in coronavirus disease 2019.

Chest radiograph	Lung POCUS
Patchy ground glass opacities	Irregular pleural line
Unable to assess peri-pleural edema	Peri-pleural edema
Dense consolidations with increasing severity of disease	Sub-pleural consolidations
Minimal to no findings possible	Diffuse B-lines
Pleural effusions rare	Minimal to absent pleural effusions
Peripheral and basal findings predominant	Posterior and basal findings predominant
